# Novel Molecular Targets Participating in Myocardial Ischemia-Reperfusion Injury and Cardioprotection

**DOI:** 10.1155/2019/6935147

**Published:** 2019-05-28

**Authors:** Nan-Bo Liu, Min Wu, Chen Chen, Masayuki Fujino, Jing-Song Huang, Ping Zhu, Xiao-Kang Li

**Affiliations:** ^1^Department of Cardiac Surgery, Guangdong Cardiovascular Institute, Guangdong Provincial People's Hospital, Guangdong Academy of Medical Sciences, Guangzhou, Guangdong 510100, China; ^2^Division of Transplantation Immunology, National Research Institute for Child Health and Development, 2-10-1 Okura, Setagaya-ku, Tokyo 157-8535, Japan; ^3^National Institute of Infectious Diseases, 1-23-1 Toyama, Shinjuku-ku, Tokyo 162-8640, Japan

## Abstract

Worldwide morbidity and mortality from acute myocardial infarction (AMI) and related heart failure remain high. While effective early reperfusion of the criminal coronary artery after a confirmed AMI is the typical treatment at present, collateral myocardial ischemia-reperfusion injury (MIRI) and pertinent cardioprotection are still challenging to address and have inadequately understood mechanisms. Therefore, unveiling the related novel molecular targets and networks participating in triggering and resisting the pathobiology of MIRI is a promising and valuable frontier. The present study specifically focuses on the recent MIRI advances that are supported by sophisticated bio-methodology in order to bring the poorly understood interrelationship among pro- and anti-MIRI participant molecules up to date, as well as to identify findings that may facilitate the further investigation of novel targets.

## 1. Introduction

Among clinical emergency events, ST-segment elevation (STE) or the non-STE electrocardiogram diagnosis of acute myocardial infarction (AMI) is particularly common worldwide, with a staggering number of annual first episodes as well as recurrent ones [1]. The most effective early treatment for reducing AMI injury and limiting the infarcted myocardium is timely coronary revascularization using thrombolytic therapy or primary percutaneous coronary intervention (PPCI) [2–4]. However, while myocardial reperfusion is well established, the process itself can trigger myocardial reperfusion injury by causing further cardiomyocyte death through multiple pathophysiological mechanisms [3–5].

**Figure 1:**
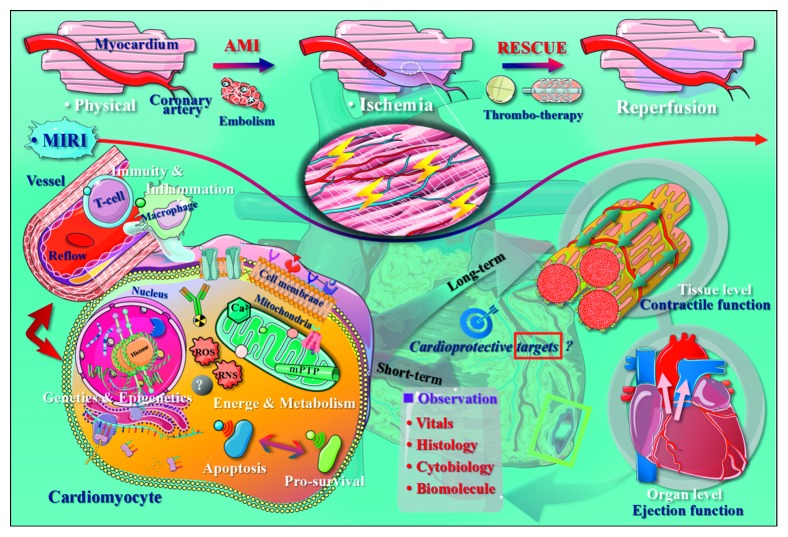
Conceptual diagram of the development and unknown mechanisms of myocardial ischemia-reperfusion injury. The pathophysiological nature of MIRI is the short-term disturbance of myocardial energy and metabolism caused by reflow after ischemia and hypoxia in the coronary artery and the dynamic changes in apoptosis and the prosurvival signaling pathways in response to related injury factors. During injury stimulation, the major effects on the cardiac function may be those involving mitochondria-dominated events along with potential nucleus-governed genetic/epigenetic alternations within the cardiomyocytes as well as the macrophage-led inflammation and T-cell-led immune responses underlying the myocardium-vessel interactive cascade. There are still many unknown aspects of MIRI's key molecular mechanisms that merit further study through both in vivo and in vitro MIRI models to discover novel functional molecular targets and identify associated cardioprotective mechanisms, which is important for improving the current treatment of AMI and MIRI. AMI, acute myocardial infarction; MIRI, myocardial ischemiareperfusion injury; ROS, reactive oxygen species; RNS, reactive nitrogen species; mPTP, mitochondrial permeability transition pore.

This coupled comorbidity of pathological ischemia and therapeutic reinjury of infarcted myocardium, namely, myocardial ischemia-reperfusion injury (MIRI), is particularly refractory to treatment [4, 5]. Traditionally, MIRI can be due to reactive oxygen and nitrogen species (ROS/RNS) generation, a reduced availability of nitric oxide (NO), Ca^2+^ overload, and mitochondrial permeability transition pore (mPTP) opening. It is important to understand how these mechanisms are dynamically regulated by pivotal molecular targets and potentially reversed in the context of cardioprotection [6]. For instance, some studies have suggested that in addition to antioxidant enzymes, nitric oxide synthases (NOSs), and other traditional enzymes, novel molecular targets such as mitochondria-targeting hydrogen sulfide (H_2_S) donor AP39 and its auxiliary targets have recently been identified as critical participants in H_2_S synthesis for modulating the postischemic cardiomyocyte survival in a manner independent of classical cytosolic signaling mechanisms [7, 8]. In addition, with the continuous advancement of the isolation and cultivation of cardiomyocytes, MIRI model establishment, multidimensional quantitation of myocardial infarct size, and improved methods for evaluating cardiomyocyte functions both *in vitro* and *in vivo* [9, 10], the subcelluar localization and mechanisms underlying the activities of novel cardioprotective genes and proteins have increasingly been discovered, with effects of diminishing cardiomyocyte apoptosis and reducing the infarct size after myocardial ischemia-reperfusion [5, 11, 12]. However, there yet remain unknown aspects of MIRI and cardioprotection, and in Figure 1, we present a briefly summarized conceptual diagram of the pathophysiology of MIRI involving the parts mentioned above.

To facilitate comprehension of the discordance between the many positive animal outcomes and the inconsistent findings of the clinical data, focusing on significant and universal cellular mechanisms contributing to MIRI in order to identify more novel cardioprotective targets will be necessary [10, 13]. Typically, post-AMI MIRI is characterized by the deformation and/or rupture of mitochondria [14], incompetency of the redox respiratory chain [15], microvascular inflammation [16], and responsive immunoreaction [17]. Damage to the myocardial mitochondria that causes disordered mitochondrial metabolism during early reperfusion is a key mechanism underlying the overlapping occurrence of itself, as well as the other three in the progression of cardiomyocyte death (Figure 1), thereby leading to a number of AMI patients with sustained substantial myocardial damage and even heart failure despite timely and successful reperfusion [2, 9, 13]. Therefore, in addition to traditional protocols, new effective strategies, including genomics, epigenetics, and proteomics, directed at novel biochemical targets for efficient cardioprotection are needed in order to limit MIRI and preserve the post-AMI cardiac function, thereby preventing the onset of heart failure and improving the patient survival [18–20].

We herein review several novel MIRI-driven agents and antagonistic targets for cardioprotection, mainly from the biochemical levels to the potential therapeutic implications.

## 2. Mitochondrial Factors of MIRI

### 2.1. Novel Cardioprotective Effects of Oxidative Stress Inhibition in MIRI

MIRI develops when the effective myocardium-supporting circulation is decreased and subsequently restored, and cardiomyocyte mitochondrial breakdown during MIRI is the common denominator stemming from the aberrant functioning of the controller that maintains homeostasis between oxidative and reductive stress, which are typically dual dynamic phases experienced by the cells adapting towards endogenous or exogenous noxious stimuli [14, 21]. In contrast, maladaptation during oxidative stress plays a critical role in the pathophysiology of MIRI. Postconditioning MIRI rats with H_2_S suggests that interfibrillar mitochondria (IFM) play an important role in cardioprotection against MIRI [22]. An exploration of the protective effect of AP39 [8], a mitochondria-specific H_2_S donor, against MIRI using substrates for complex I (glutamate and malate) and complex II (succinate, in the presence of rotenone to inhibit complex I) in both IFM and subsarcolemmal mitochondria (SSM) showed that AP39 could inhibit mito-ROS generation and mPTP opening via a cyclophilin D-independent mechanism without influencing mitochondrial respiration [8, 23]. One major source of damage underlying MIRI by ROS may be succinate-driven reverse electron transport (RET) through complex I, which is self-limiting and therefore transient [23, 24]. During MIRI, H_2_O_2_ production may be related to Mg^2+^-dependent NADH generation by malic enzyme, and it can be blocked by stigmatellin, indicating its origin from complex III, and by piericidin, demonstrating the importance of NADH-related ubiquinone in ROS reduction [24].

During myocardial ischemia, anaerobic metabolism is predominant due to energy failure, thereby reducing the intracellular pH. The Na^+^/H^+^ exchanger subsequently excretes excess hydrogen ions to buffer this accumulation of hydrogen ions, which creates a large influx of Na^+^, as well as Ca^2+^. In the process of MIRI, Ca^2+^ overload in cardiomyocyte is accompanied by the activation of intracellular proteases that damage myofibrils and induce hypercontracture and contracture band necrosis [9, 25]. Recent studies, from rodent models to human patients, have reported that succinate release by the compromised myocardium during reperfusion injury correlates with the extent of ischemia [26–28]. The selective accumulation of the citric acid cycle intermediate succinate during reperfusion is a universal metabolic signature of ischemia and contributes to mitochondrial ROS production, the crucial early driver of MIRI [26]. After reperfusion, the accumulated succinate is soon reoxidized by succinate dehydrogenase (SDH), generating extensive ROS by reverse electron transport at mitochondrial complex I [25, 26]. Based on this novel pathway underlying ROS production, the reduction of ischemic succinate accumulation and/or the inhibition of SDH can be approached as a potential therapeutic target of MIRI. For example, SDH inhibition with malonate during reperfusion has been reported to generate a reductive effect of infarcted myocardium by preventing mitochondrial permeability transition [27, 28]. However, it has recently been found that ischemic preconditioning (IPC), one of the most reproducible and robust forms of cardioprotection through a largely unknown mechanism, may not affect the accumulation of ischemic succinate or its oxidation during MIRI [29].

### 2.2. Advances in Cardioprotective Proteins in Mitochondria

Based on the hypothesis that the onset of the mitochondrial permeability transition may be a prominent mechanism in MIRI, the emerging role of mitochondrial translocator protein (TSPO) in cardioprotection has recently been targeted [30]. TSPO is a high-affinity cholesterol-binding protein that participates in mPTP formation and is associated with key cellular functions, such as apoptosis, proliferation, differentiation, and regulation of the mitochondrial function [30, 31]. Notably, 4′-chlorodiazepam, a TSPO ligand protein, protects the mitochondrial function against MIRI by inhibiting the accumulation of cholesterol and oxysterol during reperfusion, suggesting that the inhibition of cholesterol accumulation may represent a valid therapeutic strategy [32, 33]. In addition to damaging protein and lipids, ROS generated during MIRI also cause oxidative damage to DNA, inducing DNA strand breaks, nucleobase monoadducts, and covalent DNA-DNA and DNA-protein cross-links (DPCs), which can trigger DNA fragmentation and myocardial cell death [5, 34]. For example, the novel mitochondrial DNA (mtDNA) repair fusion protein exscien1-III can attenuate maladaptive remodeling during MIRI [35].

More than 60% of mitochondrial proteins contain acetylation sites involved in energy regulation, such as the inhibition of mitochondrial metabolism and ATP synthesis [36], which is critical during the process of MIRI. This type of posttranslational modification (PTM, also discussed in Section 4.2) of mitochondrial protein is mainly regulated by three SIRT family members localized in the mitochondria. The upstream roles of SIRT3 [37, 38] and SIRT5 [39] in cardioprotection against MIRI indicate potential therapeutic mitochondrial targets for treating MIRI. Concretely, through genetic rodent models with MIRI, studies have found that hearts without SIRT3 demonstrate low postischemic recovery and increased mitochondrial ROS production and mitochondrial protein oxidation [37]. Further genetic studies have identified SIRT3 as a novel mediator for cardioprotection against MIRI by stabilizing mitochondrial fission via AMPK-Drp1 pathways [38]. Similarly, SIRT5 has been identified as a pivotal factor by blocking a damage cascade through the activation of associated cytokine substrates, such as IDH-2, SDH, FUM, G6PD, ECH, and malonyl CoA, all of which in turn support oxidative phosphorylation and reduce apoptosis-inducing factor (AIF), mPTP, cytochrome c (Cyt-c), and ROS release [39, 40]. Cyt-c is a particularly important small heme-protein transferring electrons from cytc-reductase to cytc-oxidase between the inner and the outer membrane of the mitochondria [40]. Besides acetylation, MIRI-related PTMs, including phosphorylation, methylation, nitration, nitrosylation, and sulfoxidation, of several key proteins of the mitochondrial electron transport chain (ETC) are also considered potential cardioprotective targets [18, 40, 41]. For example, Cyt-c phosphorylation of Tyr97 is responsible for heart-specific phosphorylation that regulates cellular respiration, apoptosis, and ROS production and scavenging in the heart [24, 42, 43]. Regarding the role of Cyt-c in mitochondrial dysfunction, interventions designed to protect mitochondrial PTMs from ROS might be cardioprotective against MIRI.

### 2.3. Cardioprotection from Mitophagy Activation and Mitochondrial Fission Suppression

MIRI is often initiated as an adaptive response to primary ischemia injury [5, 9, 10, 13] and anticipates the tissue to meet an increased demand for oxygen [13, 21, 31]. As the heart is a highly oxidative organ, mitochondrial fission actively undertakes a dramatic role in the development of MIRI, and another critical early function of mitochondrial dynamics is the selective removal of damaged and dysfunctional mitochondria through mitochondrial autophagy, namely, mitophagy, which influences cardiomyocytes, cardiac fibroblasts, and vascular smooth muscle cells [44, 45].

Under physiological conditions, ischemia activates FUN14 domain containing 1 (FUNDC1), a receptor that mediates mitophagy caused by hypoxia and mitochondrial stress through interaction with Microtubule Associated Protein 1 Light Chain 3 Alpha (LC3), to selectively remove the damaged mitochondria and confine Cyt-c effusion, thus inhibiting cardiomyocyte apoptosis [46]. Accordingly, FUNDC1 is phosphorylated by SRC kinase and CSNK2/CK2 under normoxic conditions but dephosphorylated by PGAM5 or other unknown phosphatases under hypoxic conditions that can boost the FUNDC1-LC3 interaction [46]. During MIRI, increasing receptor-interacting serine/threonine-protein kinase 3 (RIPK3) [47] and protein kinase CK2*α* [48] inactivate the antiapoptotic effect of FUNDC1 and subsequently decrease the mitophagy of cardiac cells, thus causing a larger infarcted myocardium and cardiac/microvascular dysfunction [46–48]. An in-depth study of the mechanism suggested that Mst1 may suppress the expression of FUNDC1 via the MAPK/ERK-CREB pathway and that loss-of-function of Mst1 can generate cardioprotective effects during MIRI by preserving FUNDC1-related mitophagy; this indicates Mst1 to be a novel therapeutic regulator to fight against MIRI [49]. Through loss-of-function experiments supplementing with gain-of-function studies, more anti-MIRI targets directly associated with mitochondrial fission or mitophagy have been identified, including dynamin-related protein 1 (Drp1) [50], dual-specificity protein phosphatase 1 (DUSP1) [51], Bax inhibitor 1 (BI1) [52], and melatonin [53, 54] (e.g., summary in Table 1). Notably, ample evidence indicates that the downstream effectors of MIRI are regulators of mitophagy, and accordingly, subsequent cascade amplication of these novel targets may be controlled genetically or epigenetically [46–53]. Still, the precise upstream molecular mechanism of mitophagy remains largely unclear, and the above findings suggest potential approaches for mitochondria-targeted prevention and/or treatment of MIRI by pharmacological compounds targeting the key regulatory mechanisms of mitophagy [55].

Besides activation of mitophagy in the MIRI myocardium, inhibition of mitochondrial fission in the criminal cardiac microvasculature to control the necroptosis of cardiomyocytes is also another potential cardioprotective approach since the ongoing mitochondrial fission that escape mitophagy could abnormally disorder both function and structure of mitochondria and ultimately trigger mitochondrial destruction at the stage of reperfusion [44]. Indeed, Mitochondria fission factor (Mff) expression upregulated in response to MIRI. In contrast, Mff-knockout showed a less severity of MIRI. More noteworthy is that some vital genes and/or proteins in MIRI microvessel can be involved in the promotion of mitochondrial fission and the simultaneous inhibition of mitochondrial autophagy. For example, an actively upregulated gene termed as nuclear receptor subfamily 4 group A member 1 (NR4A1) was found to activate CK2*α*-Mf-FUNDC1 signaling axis that can generate more mitochondrial fission and less mitophagy, respectively. Superfluous mitochondrial fission generates malignant mitochondria fragmentations that cannot be removed by mitophagy, which ultimately mediates cellular death and microvascular collapse.

Further investigation of the role of BI1 in MIRI demonstrated that BI1 may intensely control the injury-echoed mitochondrial fission and mitochondrial apoptosis so as to rescue endothelial cell viability. This cardioprotective regulation mainly depends on the direct inhibition of BI1 towards the Syk pathway, which causes the downregulation of Nox2 and subsequently inhibits ROS and Drp1 phosphorylation. Another study of the cardioprotective role of melatonin shows that interaction between voltage-dependent anion channel 1 (VDAC1) and hexokinase 2 (HK2) can be recovered by melatonin and therefore blocking mitophagy-associated pathology by inhibiting mitochondrial fission through mPTP opening prevention. The decreased level of mitochondrial fission during MIRI may be associated with the inactivation of Drp1 triggered by AMPK*α*-mediated posttranscriptional modification of Drp1, and the suppression of mitochondrial fission may preserve the amount of mitochondrial HK2 by restraining VDAC1 oligomerization.

## 3. Immunoreaction of MIRI

### 3.1. Inflammation Responding Targets Involved in MIRI

Since myocardial infarction is strictly, at least to a large extent, associated with atherosclerosis, apart from the mechanical blockage of the criminal coronary vessels, ischemia plus rescuing reperfusion usually triggers local sterile inflammatory responses as well as myocardial cell apoptosis [56, 57]. However, clinically effective intervention targeting this pathophysiological process is still lacking with little understanding of its mechanisms [56, 57]. In particular, certain proinflammatory mediators and cells may exert both detrimental and protective effects, even in the same cardiac cell population, and the discovery of related critical pathways might lead to the development of more useful therapeutic strategies [58–60].

The spontaneously elevated adenosine receptor A_2B_R found in myocardium during MIRI may exert an infarct-limiting effect in the reperfused heart at an early stage not by targeting cardiomyocytes but possibly by affecting macrophages via the PI3K/Akt pathway, which is believed the vigor to switch cardiac macrophage phenotypes to an anti-inflammatory M2 subset [61–63]. Furthermore, based on the pivotal role of macrophage activation by toll-like receptors (TLRs) in the initiation of the rapid expression of proinflammatory cytokines, such as TNF*α* and IL-6, loss-of-function studies have suggested that TLR5 may play an important role in cardioprotection against MIRI [64, 65]. In brief, the loss of TLR5 markedly exacerbates MIRI, as proven by the greater cardiac and systemic expression of inflammatory cytokines, as well as larger myocardial infarcts [64]. However, the endogenous ligand of TLR5 underlying MIRI remains undetermined [64, 65].

In contrast, the spontaneously upregulated protein OPHN1 has been suggested to play a potential role as a cardioprotective inflammation regulator, which may inhibit macrophage migration and cardiomyocyte apoptosis in MIRI mice [66]. Interestingly, the PTM-stimulated endogenous accumulation of intracellular macrophage migration inhibitory factor (MIF), an important cardioprotective inflammatory cytokine released from cardiac cells and rapidly decreased during the early phase of reperfusion [67], has been reported to be able to preserve the intracellular effect of MIF for alleviating MIRI-related inflammation per se [16, 68]. However, the administration of additional recombinant MIF in mice seemingly showed no significant cardioprotective effects during the development of MIRI either *in vitro* or *in vivo*, which might reinforce the hypothesis that the direct supply of exogenous MIFs may not be beneficial in terms of infarct size reduction due to undetected mechanisms [69].

When profiling the inflammatory response mediated by macrophages, chemerin15 (C15; an endogenous anti-inflammatory component belonging to chemerin family) was identified as a novel effector that can ameliorate MIRI by decreasing cardiomyocyte apoptosis, reducing neutrophil infiltration, and, more interestingly, inducing alternative M2 macrophage polarization [70]. While a study [71] of the potential inflammatory pathways of chemerin-derived peptides/proteins suggested that the ligand-receptor interaction between chemerins and CMKLR1/GPR1 through the RhoA/ROCK pathway might be responsible, the mechanism through which C15 is involved in MIRI remains unclear [70, 71]. In addition, a study using transplantation model of M2b macrophages induced from bone marrow by LPS to treat MIRI mice revealed similar protective outcomes, suggesting that A20 (also known as TNF*α*-induced protein 3; TNFAIP3) may somehow be involved in such cardioprotection by limiting NF-*κ*B signaling-guided inflammation, although the mechanisms underlying the activation of A20 in cardiomyocytes require further investigation [72, 73]. The I*κ*B kinase IKK*ɑ* has also been identified as a novel mediator capable of attenuating the outcomes of MIRI, possibly by the negative control of macrophage polarization towards the M1 phenotype [57].

Another novel component of the acute inflammatory response in the process of MIRI is nucleotide-binding oligomerization domain-like receptor (NLR) family [74–76], an upregulated member of which in the mice model is NOD2 that may aggravate MIRI through JNK/p38MAPK/NF-*κ*B signaling [77, 78]. In contrast, NOD2 deficiency generated by overexpressing the negative regulator TIPE2 can reduce the levels of proinflammatory mediators and cardiac inflammatory cell infiltration after ischemia/reperfusion [79, 80]. Taken together, these previous findings suggest that downregulating NOD2 is a potential therapeutic approach worth further research in clinical translation, and multilevel targeting of NOD2-mediated TIPE2 signaling pathways may provide a novel treatment for MIRI as well as other cardiovascular diseases [74–79]. Furthermore, these studies (as shown in Table 2) also provide noteworthy evidence supporting our understanding of the regulatory mechanisms of the protein interactions associated with the inflammatory network in the cardiovascular system, and therapeutic approaches targeting specific components of the inflammatory response are promising [56, 81].

### 3.2. Alternative Immunoresponsive Substrates Inducing MIRI

Following the early inflammation stage, pathophysiological changes in MIRI might next cause endogenous signals that may trigger anti-inflammatory phases dominated by the innate immune response [82, 83], the transitional stage of which may involve a sophisticated interaction between cardiac cells and components of the immune response [84, 85]. Since the cellular immune response and inflammatory microenvironment can, to a large extent, trigger adverse cardiac remodeling in MIRI patients [84, 86]; researchers and clinicians have been trying to identify potential therapeutic molecular and cellular targets for AMI from the perspective of both the innate and adaptive immune systems [85, 87]. For example, through *in vitro* and *in vivo* experiments, several protective cell types of the immune system have been found to actively respond to MIRI, including pericytes, Ly6C^high^ monocytes, M2 macrophages, and CD8^+^/AGTR2^+^ T cells [85–89].

Increasing evidence support the role and potential therapeutic value of regulatory T lymphocytes (Tregs, an essential CD4^+^/CD25^+^ subset of lymphocytes) in fighting MIRI [89–91], although the comprehensive and precise mechanism involved remains unknown. In detail, Tregs attenuate cardiomyocyte apoptosis by activating the Akt and ERK1/2 pathways and reduce neutrophil infiltration via the downregulation of the production of cytokine-induced neutrophil chemoattractant (CK) and lipopolysaccharide-induced CXC chemokine production (LIX), the triggering mechanism of which might be CD39-dependent [92]. In this prosurvival pathway, CD39 represents a potential inhibitory mechanism for recruited Tregs to suppress neutrophil infiltration and chemokine production so as to maintain cardiomyocyte viability in MIRI hearts [92]. Indeed, Tregs recruitment in MIRI currently stands out in the immunity-driven cardioprotection against MIRI.

Additionally, several recent studies have suggested that Tregs may antagonize myocardial remodeling and improve the mechanical function of the MIRI heart via epicardial Hippo signaling (epicardial YAP/TAZ can recruit Tregs to injured myocardium after MI) [93], paracrine effects [93, 94], and interactions with the IL-2/Anti-IL-2 complex [95]. Notably, endogenous Tregs could preserve heart function after AMI by regulating IL-2 related inflammation and collagen homeostasis [93, 94], and exogenous Tregs might sustain cardiomyocyte proliferation without using their typical immunosuppressive ability [93, 94]. It is the IL-2C-mediated CD25 rather than IL-10 or TGF-b1 that drives Tregs to expand and attenuate MIRI by maintaining cardiomyocyte's viability. Another investigation showed that, when applied at an early stage of AMI, the Tregs recruited by *N*, *N*-dimethylsphingosine may regulate protection against MIRI via the inhibition of the innate immune response through the PI3K/Akt pathway [96]. A study on diabetic patients with STEMI (routine treatment versus routine treatment plus the drug vildagliptin) showed that the addition of vildagliptin (an oral antihyperglycemic agent) to standard medical treatment of MIRI increased its effectiveness via the recruitment of Tregs by significantly upregulating TGF-*β*1 [97]. Therefore, from a regeneration perspective, clarifying whether or not myocardium regeneration can be promoted in an MIRI model merely by regulating the concentration of Tregs *in situ* appears to be a promising direction [98, 99].

As immunotherapy of post-MIRI patients has been actively explored, a recent study regarding sphingosine-1 phosphate (S1P) and its analogue FTY720 showed that postconditioning with FTY720 was able to mitigate MIRI in patients as effectively as splenectomy but with improved long-term outcomes [100]. One possible mechanism of S1P/FTY720-induced cardioprotection against MIRI, as studied in rats, involves the inhibition of glycogen synthase kinase (GSK)-3*β* as well as the regulation of mPTP [101]. Similarly, immunosuppression with FTY720 can prevent postinfarction myocardial remodeling and chronic heart failure by reducing immune B cells and the associated chemokine CCL7 as well as by suppressing the responsive expression of MMP-2 and IL-6, thereby preventing the heart from developing severe cardiac inflammation and inducing an immune response [102]. Intriguingly, an investigation using whole-genome screening for potential FTY720-responsive mutants surprisingly carries out genes required for ROS homeostasis [103], encouraging the investigation of the potentially important relationship between immune-related mediators and ROS-guided pathological changes, such as MIRI.

Other emerging targets of autoimmune response in MIRI treatment include TRAF3 interacting protein 2 (TRAF3IP2; an oxidative stress-responsive cytoplasmic adaptor molecule that is an upstream regulator of both IKK and JNK) [104, 105], pleiotropic cytokine IL-21 promoting MIRI via the modulation of neutrophil infiltration [106], adaptor protein Crk mediating T-cell adhesion [107], and adenosine with its functional receptors [62, 108]. Since the immune network might exert both cardioprotective and cardiopathic effects during MIRI (as shown in Table 3), studies on novel therapeutic approaches that target the control of immune system, including the above-mentioned targets, to result in more balanced inflammatory and immune responses upon MIRI are still warranted [76, 84, 109].

## 4. Genetics and Epigenetics of MIRI

### 4.1. Prominent Genetic Loci Associated with MIRI

To investigate the mechanisms underlying potentially crucial molecular targets in MIRI for corresponding treatment, their expression in the myocardium at a genome-wide level must be determined [84, 110, 111]. Because of the complexity of MIRI-related pathophysiology, especially the diversity of potential sequence variants as well as the detectable epigenetic modifications that may alter the gene expression profiles among different participating cell populations [9, 10, 13], it is conceivable that genome biological techniques, such as high-throughput and high-resolution gene sequencing, might be more successful for detecting unidentified therapeutic targets and therefore better understanding the signaling network responsible for MIRI and cardioprotection [84, 110]. A transcriptional regulation study through bioinformatics revealed that four potential hypoxia-inducible factor-1 alpha (HIF-1*α*)-binding motifs on the promoter of renalase (an enzyme elevated during MIRI that can metabolize catecholamine) are novel loci involved in cardioprotection against MIRI [112]. S100A1 [113] and S100A6 [11], members of the S100 family protein containing EF-hand and Ca^2+^-binding motifs, as well as Annexin-5 (a member of the Annexin family containing Ca^2+^-dependent phospholipid binding motifs) [12], might regulate several intrinsic apoptosis pathways involved in MIRI, generating a smaller infarct size and improving the systolic function of the left ventricle. Similarly, the innovative roles and action sites of several cardioprotective genes against MIRI associated with intracellular homeostasis and antiapoptosis have been signposted using bioinformatic methods, such as STAT3 [114], GSTP [115], SGLT1 [116], BCL2 [117], and SERCA2 [118].

MIRI can also genetically activate intracellular signaling pathways, like mitogen-activated protein kinases (MAPKs), emerging subfamilies of which include p38MAPK and JNK that might trigger the inflammation-apoptosis chain during MIRI by regulating the expression of extracellular regulated kinase (ERK1/2) [119, 120]. Indeed, the detection of the activity of p38MAPK and JNK has been currently designed as a paradigm for estimating the cardioprotective effects against MIRI of the developing therapeutic molecules (e.g., morphine [121], kaempferol [122], febuxostat [123], and triiodothyronine [124]) in the very dimension of gene expression. Accordingly, the overexpression of the upstream downregulatory genes of the MAPK pathway (e.g., DUSP1 [51], DUSP14 [125], and RGS5 [126]) and/or silencing of its up-regulatory genes (e.g., FPR1 [127]) might be useful for preventing MIRI by suppressing cardiac inflammation, cardiomyocyte apoptosis, and ventricular remodeling [126–128].

When starting MIRI therapy with rapamycin (another therapeutic molecule with mTOR inhibition), the transcription of ERK1/2 was surprisingly found to have increased, but with p38 deactivation, and subsequent genetic evidence which confirmed the selective activation of ERK with the parallel inhibition of p38 (via the MAPK and PI3K-Akt signaling pathways) as a novel therapeutic strategy [129]. Another study regarding small molecule agonists of formyl peptide receptors (FPRs) in MIRI also suggested that the activation of ERK with concurrent stimulation of Akt (serine/threonine) kinases in the tortured heart may be an alternative therapeutic approach, additionally supporting the notion that Akt activation might be a cardioprotective event in the context of MIRI [130, 131]. In contrast, the inhibition of Akt expression blocks the cardioprotection regulated by angiotensin (a crucial peptide hormone in the cardiovascular system) both *in vitro* and *ex vivo* [132], suggesting that AT2R functioning as a receptor for angiotensin II may mediate Akt activation during cardioprotection against MIRI. However, despite this influx of recent findings, much remains unclear regarding the genetically active loci of the genome associated with MIRI and cardioprotection [110, 111].

### 4.2. Noteworthy Epigenetic Regulators Underlying MIRI

Epigenetics refers to the study of cellular heritable modifications involving PTM (e.g., methylation/demethylation and acetylation/deacetylation) of histone or nonhistone protein, DNA methylation, and chromatin architecture without changing its DNA sequence, which is considered an important reader-writer-eraser mechanism in gene expression in response to rapid and drastic events, such as cardiovascular injury or disease [9, 133, 134]. Since MIRI is a progressive attack with cumulative pathophysiological epigenetic changes that might be integrated to impair the heart function [135], the major molecular substrate dominating persistent heart dysfunction should also be investigated in order to develop next-generation therapeutic targets [135, 136]. To such ends, emerging well-verified evidence over the last decade has begun showing important traces of the epigenetic regulators dominating in MIRI and cardioprotection [134–137].

**Figure 2:**
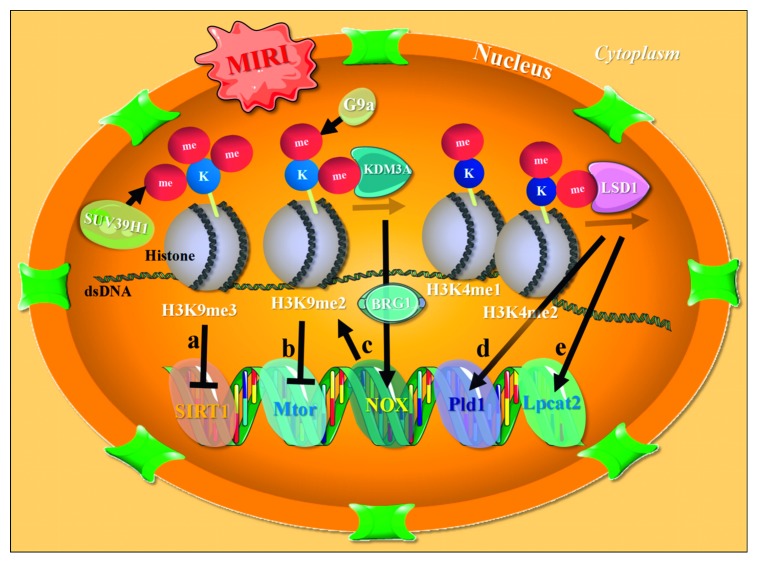
Potential regulation of histone methylation/demethylation underlying MIRI. (a) MIRI can cause increased cardiac H3K9me3 at the proximal SIRT1 promoter through responsive SUV39H1, which may subsequently inhibit the transcription of SIRT1. (b) Increased H3K9me2 post-IP is G9a-dependent and potentially suppresses Mtor and other MIRI responsive genes. (c) KDM3A demethylates H3K9 at the NOX promoter and interacts with BRG1 in order to activate NOX transcription, the suppression of which is paralleled by the local reappearance of H3K9me2. (d, e) The LSD1-guided demethylation of the promoter H3K4me1/2 of Pld1 and Lpcat2 may be cardioprotective against MIRI. dsDNA, double-stranded deoxyribonucleic acid; H3K9me3, trimethylation of lysine 9 of histone 3; H3K9me2, bimethylation of lysine 9 of histone 3; H3K4me1, monomethylation of lysine 4 of histone 3; H3K4me2, bimethylation of lysine 4 of histone 3.

In a recent study of the epigenetics of MIRI, the role of the nicotinamide adenine dinucleotide- (NAD^+^-) dependent and strictly conserved protein deacetylase family (SIRT1-7) has been well studied [138]. The upregulation of SIRT1 back to the physiological level can block the death process of cardiac cells at distinct key points of MIRI, possibly by deacetylating HSF-1 in order to increase the HSP70/HSP90 expression [139]. Based on their findings, those authors suggested that the maintenance of SIRT1 expression might be a potential approach for treating MIRI [139], while a later study reported that upregulating cardiac SIRT1 in a mouse MIRI model by exogenous H_2_S postconditioning was able to improve the cardiac function and reduce the infarct size via the PGC-1*α* signaling pathway [140]. Furthermore, findings from subsequent *in vitro* and *in vivo* experiments suggested that the natural accumulation of trimethylation of lysine 9 of histone 3 (H3K9me3; a repressive histone modification) at the proximal SIRT1 promoter by SUV39H1 (a H3K9 tri-methyltransferase) in myocardium suffering from MIRI might be the epigenetic mechanism responsible for the downregulation of SIRT1 in this scenario (Figure 2(a)) [141–143]. Mitochondrial SIRT3, as mentioned in Section 2.2 above, is a novel cardioprotector against MIRI [37, 38], functioning mainly through enhancing the deacetylation of cyclophilin D via the upregulation of SIRT3 in order to stop the formation of excessive mPTP and prevent subsequent cell death [144, 145]. Likewise, SIRT5 may function as a promising novel therapeutic target for MIRI through epigenetically modifying the substrates associated with mitochondrial dynamics and oxidative phosphorylation [39, 146]. However, despite recent developments in histone deacetylase- (HDAC-) guiding pharmacology, such as HDAC inhibition, for MIRI treatment [147, 148] and the specific and beneficial epigenetic mechanisms of MIRI per se and cardioprotection, such as the regulation of methylation/demethylation of histone and nonhistone substrates, remain largely unknown [137, 138, 149].

Evidence also suggests that methylation/demethylation of histone may be crucial for the treatment of MIRI, as well as for the further study of cardioprotection. In a study exploring the epigenetic response mechanism of cardiac IPC [150], researchers surprisingly found that over 200 genes were transcriptionally repressed with the enrichment of the bimethylation of lysine 9 of histone 3 (H3K9me2; another repressive histone modification) in the myocardium processed with IPC. Subsequent mechanistic studies confirmed that this increase in H3K9me2 levels was G9a-dependent and potentially regulated cardiac autophagy by suppressing MIRI-responsive genes, such as Mtor (Figure 2(b)) [150, 151]. Another study [152] on myocardial lysine-specific demethylase 1 (LSD1), an important histone H3 demethylase capable of eliminating mono- and dimethyl groups through oxidative cleavage, suggested that the LSD1-guided demethylation of the promoter H3K4me1/2 of the downstream genes (e.g., Pld1 and Lpcat2 shown in Figure 2 (d, e) might play an essential role in cardioprotection underlying MIRI [152].

Another recent study [153] characterizing the chromatin remodeling protein BRG1 in response to cardiac hypoxia-reoxygenation *in vitro* showed that the H3K9 demethylase KDM3A binding to NOX promoter interacted with BRG1 to activate NOX transcription, the suppression of which was paralleled by the local reappearance of H3K9me2 (Figure 2(c)) as well as the loss of active markers, such as H3 and H4 acetylation. Furthermore, subsequent *in vivo* MIRI experiments [154] have shown that MIRI downregulates the levels of H3K9me3 at the PODXL promoter and that the knockdown of BRG1 can restore H3K9me3, while the transactivation of PODXL by the BRG1-KDM4B complex *in situ* promotes neutrophil infiltration and exacerbates MIRI. These well-described findings suggest the switching of gene profiling via the regulation of key epigenetic enzymes might be practical for the study and treatment of MIRI and cardioprotection [153, 154]. Further exploration of the causality between histone modification and MIRI (e.g., at the single-cell level) to develop more novel therapeutic targets is, however, still warranted.

## 5. Conclusions

Despite early reperfusion into the coronary artery with PPCI and/or other advanced treatment, the clinical morbidity and mortality of MIRI remain significant [2, 5, 23, 84] due to the multidimension and spatiotemporal complexity of its pathophysiologic determinant [9, 10, 84]. With the rapid progress of biomedical research in the past decade, such as the development of gene-editing technology and animal models, dynamic confocal microscopy imaging, and ChIP-seq and bioinformatic analyses of large data, the mechanisms underlying MIRI have already begun to be revealed by researchers [25, 34, 134, 135]. From the regulation of the inflammatory-immune response [76, 79–82] and the energy and metabolism in mitochondria [39–44] to the epigenetic modification of chromatin [134–137], more and more novel molecular targets for MIRI and cardioprotection are being identified. Over time, more beneficial findings regarding the leading edge of MIRI and cardioprotection will hopefully mature and be applied in clinic to benefit more AMI patients. However, before that, more rigorous and innovative *in vivo* and *in vitro* protocols must be proposed in order to resolve all scientific issues concerning the MIRI mechanism and beyond [10]. In spite of the promising indication of the innovative findings of a set of novel molecular agents participating in various biological processes against MIRI from basic experimental studies as previously highlighted, and a worldwide intensification of grants into the research in this field, there is yet an effective therapeutic target or drug to treat MIRI patients [2, 4]. One crucial reason, as mentioned above, might be that MIRI is usually multifactorial and triggers cardiomyocyte death via diverse synergistic mechanisms and multiple cell types throughout the pathological transformation. In this regard, an obedience to certain preclinical recommendations and guidelines that have been recently put forward for MIRI's multitarget strategies will be necessary for its basic studies from bench to bedside so that the critical bottlenecks towards clinical translation of cardioprotection against MIRI can be effectually resolved [5, 9, 155]. Nonetheless, accumulated data from the findings mentioned in this review might facilitate the further identification of novel targets involved in MIRI and cardioprotection.

## Figures and Tables

**Table 1:** The fresh mitochondria-targeted episodes underlying MIRI.

Model	Effector	Target	Activity in MIRI	Reference
Mouse	Rotenone, MitoSNO8	Complex I RET protein	Inhibit complex I RET to abolish ischemic succinate and dimethyl succinate-driven DHE oxidation	[26]
	Malonate	SDH	Inhibits SDH to block mitochondrial permeability transition	[27]
	Exscien1-III	mtDNA sequence	Increases mitochondrial antioxidant and apoptotic markers	[35]
	SIRT3	AMPK-drp1	Inhibits excessive mitochondrial fission and normalize AMPK-Drp1 pathways	[38]
	SIRT5	IDH-2, SDH, FUM, G6PD	Inhibits calcium overload, AIF, mPTP opening, ROS and Cyt-c release	[39]
	FUNDC1	LC3, Ripk3, CK2*α*, Mst1	Stabilizes mitophagy to inhibit cardiomyocyte apoptosis	[46–49]
	DUSP1	Mff, Bnip3	Inactivates the JNK pathway to alleviate the fatal mitochondrial fission/mitophagy	[51]
	BI1	F-actin	Inhibits mitochondrial fission through the XO/ROS/F-actin pathways	[52]
	Melatonin	PGAM5, Ripk3	Inhibits mitochondrial fission and necroptosis through Ripk3-PGAM5-CypD-mPTP pathways	[53]

Rat	H_2_S	AP39	Inhibits mito-ROS generation and mPTP opening	[8, 22]
	4′-chlorodiazepam	TSPO	Inhibits cholesterol and oxysterol accumulation during reperfusion	[33]
	Drp1K38A	Drp1	Decreases the oxygen-dependent metabolism	[50]

Bovine	Insulin	Tyr97	Phosphorylated by Cyt-c and in turn limits Cyt-c release and apoptosis	[41]

Human	Cyclosporin A	mPTP components	Inhibits cyclophilin D and mPTP opening	[23]

**Table 2:** Specific function of inflammation in MIRI and cardioprotection.

Model	Effector	Target	Activity in MIRI	Reference
Mouse	PI3K	Erk, Akt, GSK3*β*	Mediates the protective effect by phosphorylation during the IPC trigger phase	[63]
	BAY 60-6583	A_2B_R	Modulates proinflammatory kinases via the PI3K/Akt pathway in cardiac M2 macrophage	[61]
	TLR5	Unknown	Deficiency of TLR5 aggravates inflammation	[64]
	OPHN1	RhoA, Rac1, Cdc42	Deficiency of OPHN1 increases inflammatory cell migration and cardiomyocyte apoptosis	[66]
	S-Nitrosation	MIF	Stimulates an overall enhanced protective effect	[67]
	Chemerin15	Unknown	Decreases TNF*α* and IL-6 levels and increases IL-10 levels	[70]
	A20	Unknown	Reduces cardiomyocyte necrosis and apoptosis	[72]
	IKK*ɑ*	Unknown	Causes negative control of macrophage polarization towards M1 phenotype	[57]
	TIPE2	NOD2	Reduces the levels of proinflammatory mediators and cardiac inflammatory cell infiltration	[79]

**Table 3:** Recent recognitions upon potential MIRI-related immunity.

Model	Effector	Target	Activity in MIRI	Ref.
Mouse	Tregs	Epicardial YAP/TAZ	The novel Hippo signaling effectors YAP/TAZ within epicardial can drive the immune chemokine target IFN-γ of Tregs to the injured myocardium and function as cardioprotectors post-AMI.	[93]
	CD39 of Tregs	Unknown	Attenuates cardiomyocyte apoptosis and reduces neutrophil infiltration.	[92]
	Key secreted proteins of Tregs	Unknown	Tregs function in a paracrine manner to promote cardiomyocyte proliferation for cardioprotection after AMI. The six secreted proteins including Cst7, Tnfsf11, Il33, Fgl2, Matn2, and Igf2 may be responsible.	[94]
	IL-2/Anti-IL-2 mAb complex (IL-2C)	Unknown	IL-2C from the spleen and heart might selectively proliferate cardioprotective Tregs.	[95]
	*N*,*N*-dimethylsphingosine (DMS)	IL-10, TGF*β*	DMS applied during early AMI *in vivo* may be protective against MIRI by recruiting Tregs via the PI3K/Akt pathway.	[96]
	S1P/FTY720	CCL7, MMP-2 and IL-6	FTY720 can reduce immune B cells and its associated chemokine CCL7 and suppress MMP-2 and IL-6 in order to prevent the heart from severe cardiac inflammation and immune responses.	[102]
	TRAF3IP2 (previously known as CIKS or Act1)	NF-*κ*B, JNK, p38 MAPK	*Traf3ip2* gene deletion mediates an overall cardioprotective effects.	[104, 105]
	IL-21	Akt, NF-*κ*B, p38MAPK	Increases chemokine expression by activating Akt/NF-*κ*B signaling in cardiomyocytes and p38 MAPK/NF-*κ*B signaling in cardiac fibroblasts.	[106]
	Crk adaptor proteins	C3G, RAP1	Crk adaptor proteins can mediate the initial steps of T-cells adhesion via its nSH3 domain binding to C3G, which is guanine-nucleotide exchange factors for the small GTPases RAP1.	[107]

Rat	S1P/FTY720	(GSK)-3*β*, mPTP components	S1P receptor agonist FTY720 can inhibit GSK-3*β* and regulate opening of mPTP to be cardioprotective.	[101]

Human	Vildagliptin	TGF-*β*1	Vildagliptin can recruit Tregs by overexpressing TGF-*β*1.	[97]

## Abbreviations

AMI: Acute myocardial infarction
MIRI: Myocardial ischemia-reperfusion injury
STE: ST-segment elevation
RET: Reverse electron transport
PPCI: Primary percutaneous coronary intervention
MIRI: Myocardial ischemia-reperfusion injury
NO: Nitric oxide
ROS: Reactive oxygen species
RNS: Reactive nitrogen species
mPTP: Mitochondrial permeability transition pore
H_2_S: Hydrogen sulfide
NOSs: Nitric oxide synthases
SDH: Succinate dehydrogenase
TSPO: Mitochondrial translocator protein
mtDNA: Mitochondrial DNA
IPC: Ischemic preconditioning
PTM: Posttranslational modification
AIF: Apoptosis-inducing factor
ETC: Electron transport chain
ERK: Extracellular regulated kinase
NAD+: Nicotinamide adenine dinucleotide
H3K9me3: Trimethylation of lysine 9 of histone 3
H3K9me2: Bimethylation of lysine 9 of histone 3
H3K4me1: Monomethylation of lysine 4 of histone 3
H3K4me2: Bimethylation of lysine 4 of histone 3
HDAC: Histone deacetylase.

## References

[1] Anderson J. L., Morrow D. A. (2017). Acute myocardial infarction. *New England Journal of Medicine*.

[2] Bainey K. R., Armstrong P. W. (2014). Clinical perspectives on reperfusion injury in acute myocardial infarction. *American Heart Journal*.

[3] Patil K. D., Halperin H. R., Becker L. B. (2015). Cardiac arrest. *Circulation Research*.

[4] Yellon D. M., Hausenloy D. J. (2007). Myocardial reperfusion injury. *New England Journal of Medicine*.

[5] Hausenloy D. J., Yellon D. M. (2013). Myocardial ischemia-reperfusion injury: a neglected therapeutic target. *Journal of Clinical Investigation*.

[6] Pagliaro P., Moro F., Tullio F., Perrelli M.-G., Penna C. (2011). Cardioprotective pathways during reperfusion: focus on redox signaling and other modalities of cell signaling. *Antioxidants and Redox Signaling*.

[7] Andreadou I., Iliodromitis E. K., Rassaf T., Schulz R., Papapetropoulos A., Ferdinandy P. (2015). The role of gasotransmitters NO, H2S and CO in myocardial ischaemia/reperfusion injury and cardioprotection by preconditioning, postconditioning and remote conditioning. *British Journal of Pharmacology*.

[8] Karwi Q. G., Bornbaum J., Boengler K. (2017). AP39, a mitochondria-targeting hydrogen sulfide (H2S) donor, protects against myocardial reperfusion injury independently of salvage kinase signalling. *British Journal of Pharmacology*.

[9] Eltzschig H. K., Eckle T. (2011). Ischemia and reperfusion—from mechanism to translation. *Nature Medicine*.

[10] Botker H. E., Hausenloy D., Andreadou I. (2018). Practical guidelines for rigor and reproducibility in preclinical and clinical studies on cardioprotection. *Basic Research in Cardiology*.

[11] Mofid A., Newman N. S., Lee P. J. (2017). Cardiac overexpression of S100A6 attenuates cardiomyocyte apoptosis and reduces infarct size after myocardial ischemia-reperfusion. *Journal of the American Heart Association*.

[12] de Jong R. C. M., Pluijmert N. J., de Vries M. R. (2018). Annexin A5 reduces infarct size and improves cardiac function after myocardial ischemia-reperfusion injury by suppression of the cardiac inflammatory response. *Scientific Reports*.

[13] Heusch G., Gersh B. J. (2017). The pathophysiology of acute myocardial infarction and strategies of protection beyond reperfusion: a continual challenge. *European Heart Journal*.

[14] Lesnefsky E. J., Chen Q., Tandler B., Hoppel C. L. (2017). Mitochondrial dysfunction and myocardial ischemia-reperfusion: implications for novel therapies. *Annual Review of Pharmacology and Toxicology*.

[15] Granger D. N., Kvietys P. R. (2015). Reperfusion injury and reactive oxygen species: the evolution of a concept. *Redox Biology*.

[16] Ong S.-B., Hernández-Reséndiz S., Crespo-Avilan G. E. (2018). Inflammation following acute myocardial infarction: multiple players, dynamic roles, and novel therapeutic opportunities. *Pharmacology and Therapeutics*.

[17] Swirski F. K., Nahrendorf M. (2018). Cardioimmunology: the immune system in cardiac homeostasis and disease. *Nature Reviews Immunology*.

[18] Sluijter J. P., Condorelli G., Davidson S. M. (2014). Novel therapeutic strategies for cardioprotection. *Pharmacology and Therapeutics*.

[19] Altamirano F., Wang Z. V., Hill J. A. (2015). Cardioprotection in ischaemia-reperfusion injury: novel mechanisms and clinical translation. *Journal of Physiology*.

[20] Garrido V., Mendoza-Torres E., Riquelme J. A. (2017). Novel therapies targeting cardioprotection and regeneration. *Current Pharmaceutical Design*.

[21] Kurian G. A., Rajagopal R., Vedantham S., Rajesh M. (2016). The role of oxidative stress in myocardial ischemia and reperfusion injury and remodeling: revisited. *Oxidative Medicine and Cellular Longevity*.

[22] Banu S. A., Ravindran S., Kurian G. A. (2016). Hydrogen sulfide post-conditioning preserves interfibrillar mitochondria of rat heart during ischemia reperfusion injury. *Cell Stress and Chaperones*.

[23] Morciano G., Giorgi C., Bonora M. (2015). Molecular identity of the mitochondrial permeability transition pore and its role in ischemia-reperfusion injury. *Journal of Molecular and Cellular Cardiology*.

[24] Korge P., Calmettes G., John S. A., Weiss J. N. (2017). Reactive oxygen species production induced by pore opening in cardiac mitochondria: the role of complex III. *Journal of Biological Chemistry*.

[25] Neri M., Riezzo I., Pascale N., Pomara C., Turillazzi E. (2017). Ischemia/reperfusion injury following acute myocardial infarction: a critical issue for clinicians and forensic pathologists. *Mediators of Inflammation*.

[26] Chouchani E. T., Pell V. R., Gaude E. (2014). Ischaemic accumulation of succinate controls reperfusion injury through mitochondrial ROS. *Nature*.

[27] Valls-Lacalle L., Barba I., Miró-Casas E. (2016). Succinate dehydrogenase inhibition with malonate during reperfusion reduces infarct size by preventing mitochondrial permeability transition. *Cardiovascular Research*.

[28] Kohlhauer M., Dawkins S., Costa A. S. H. (2018). Metabolomic profiling in acute ST-segment-elevation myocardial infarction identifies succinate as an early marker of human ischemia-reperfusion injury. *Journal of the American Heart Association*.

[29] Pell V. R., Spiroski A.-M., Mulvey J. (2018). Ischemic preconditioning protects against cardiac ischemia reperfusion injury without affecting succinate accumulation or oxidation. *Journal of Molecular and Cellular Cardiology*.

[30] Paradis S., Leoni V., Caccia C., Berdeaux A., Morin D. (2013). Cardioprotection by the TSPO ligand 4′-chlorodiazepam is associated with inhibition of mitochondrial accumulation of cholesterol at reperfusion. *Cardiovascular Research*.

[31] Morin D., Musman J., Pons S., Berdeaux A., Ghaleh B. (2016). Mitochondrial translocator protein (TSPO): from physiology to cardioprotection. *Biochemical Pharmacology*.

[32] Musman J., Paradis S., Panel M. (2017). A TSPO ligand prevents mitochondrial sterol accumulation and dysfunction during myocardial ischemia-reperfusion in hypercholesterolemic rats. *Biochemical Pharmacology*.

[33] Thackeray J. T., Hupe H. C., Wang Y. (2018). Myocardial inflammation predicts remodeling and neuroinflammation after myocardial infarction. *Journal of the American College of Cardiology*.

[34] Groehler A., Kren S., Li Q. (2018). Oxidative cross-linking of proteins to DNA following ischemia-reperfusion injury. *Free Radical Biology and Medicine*.

[35] Bradley J. M., Li Z., Organ C. L. (2018). A novel mtDNA repair fusion protein attenuates maladaptive remodeling and preserves cardiac function in heart failure. *American Journal of Physiology-Heart and Circulatory Physiology*.

[36] Baeza J., Smallegan M. J., Denu J. M. (2016). Mechanisms and dynamics of protein acetylation in mitochondria. *Trends in Biochemical Sciences*.

[37] Parodi-Rullan R. M., Chapa-Dubocq X., Rullan P. J., Jang S., Javadov S. (2017). Corrigendum: high sensitivity of SIRT3 deficient hearts to ischemia-reperfusion is associated with mitochondrial abnormalities. *Frontiers in Pharmacology*.

[38] Liu J., Yan W., Zhao X. (2019). Sirt3 attenuates post-infarction cardiac injury via inhibiting mitochondrial fission and normalization of AMPK-Drp1 pathways. *Cellular Signalling*.

[39] Zou R., Shi W., Tao J. (2018). SIRT5 and post-translational protein modifications: a potential therapeutic target for myocardial ischemia-reperfusion injury with regard to mitochondrial dynamics and oxidative metabolism. *European Journal of Pharmacology*.

[40] Boylston J. A., Sun J., Chen Y., Gucek M., Sack M. N., Murphy E. (2015). Characterization of the cardiac succinylome and its role in ischemia-reperfusion injury. *Journal of Molecular and Cellular Cardiology*.

[41] Kalpage H. A., Bazylianska V., Recanati M. A. (2018). Tissue-specific regulation of cytochrome c by post-translational modifications: respiration, the mitochondrial membrane potential, ROS, and apoptosis. *The FASEB Journal*.

[42] Ascenzi P., Coletta M., Wilson M. T. (2015). Cardiolipin-cytochromeccomplex: switching cytochromecfrom an electron-transfer shuttle to a myoglobin- and a peroxidase-like heme-protein. *IUBMB Life*.

[43] Jenkins C. M., Yang K., Liu G., Moon S. H., Dilthey B. G., Gross R. W. (2018). Cytochromecis an oxidative stress-activated plasmalogenase that cleaves plasmenylcholine and plasmenylethanolamine at thesn-1 vinyl ether linkage. *Journal of Biological Chemistry*.

[44] Vásquez-Trincado C., García-Carvajal I., Pennanen C. (2016). Mitochondrial dynamics, mitophagy and cardiovascular disease. *Journal of Physiology*.

[45] Zhang W., Chen C., Wang J., Liu L., He Y., Chen Q. (2018). Mitophagy in cardiomyocytes and in platelets: a major mechanism of cardioprotection against ischemia/reperfusion injury. *Physiology*.

[46] Zhang W., Siraj S., Zhang R., Chen Q. (2017). Mitophagy receptor FUNDC1 regulates mitochondrial homeostasis and protects the heart from I/R injury. *Autophagy*.

[47] Zhou H., Zhu P., Guo J. (2017). Ripk3 induces mitochondrial apoptosis via inhibition of FUNDC1 mitophagy in cardiac IR injury. *Redox Biology*.

[48] Zhou H., Zhu P., Wang J., Zhu H., Ren J., Chen Y. (2018). Pathogenesis of cardiac ischemia reperfusion injury is associated with CK2*α*-disturbed mitochondrial homeostasis via suppression of FUNDC1-related mitophagy. *Cell Death and Differentiation*.

[49] Yu W., Xu M., Zhang T., Zhang Q., Zou C. (2019). Mst1 promotes cardiac ischemia-reperfusion injury by inhibiting the ERK-CREB pathway and repressing FUNDC1-mediated mitophagy. *Journal of Physiological Sciences: JPS*.

[50] Zepeda R., Kuzmicic J., Parra V. (2014). Drp1 loss-of-function reduces cardiomyocyte oxygen dependence protecting the heart from ischemia-reperfusion injury. *Journal of Cardiovascular Pharmacology*.

[51] Jin Q., Li R., Hu N. (2018). DUSP1 alleviates cardiac ischemia/reperfusion injury by suppressing the Mff-required mitochondrial fission and Bnip3-related mitophagy via the JNK pathways. *Redox Biology*.

[52] Zhou H., Wang J., Hu S., Zhu H., Toan S., Ren J. (2019). BI1 alleviates cardiac microvascular ischemia-reperfusion injury via modifying mitochondrial fission and inhibiting XO/ROS/F-actin pathways. *Journal of Cellular Physiology*.

[53] Zhou H., Li D., Zhu P. (2018). Inhibitory effect of melatonin on necroptosis via repressing the Ripk3-PGAM5-CypD-mPTP pathway attenuates cardiac microvascular ischemia-reperfusion injury. *Journal of Pineal Research*.

[54] Lochner A., Marais E., Huisamen B. (2018). Melatonin and cardioprotection against ischaemia/reperfusion injury: what’s new? A review. *Journal of Pineal Research*.

[55] Abdelwahid E., Stulpinas A., Kalvelyte A. (2017). Effective agents targeting the mitochondria and apoptosis to protect the heart. *Current Pharmaceutical Design*.

[56] Hernandez-Resendiz S., Chinda K., Ong S.-B., Cabrera-Fuentes H., Zazueta C., Hausenloy D. J. (2018). The role of redox dysregulation in the inflammatory response to acute myocardial ischaemia-reperfusion injury-adding fuel to the fire. *Current Medicinal Chemistry*.

[57] Cao Y., Xu Y., Auchoybur M. L. (2018). Regulatory role of IKK*ɑ* in myocardial ischemia/reperfusion injury by the determination of M1 versus M2 polarization of macrophages. *Journal of Molecular and Cellular Cardiology*.

[58] Frangogiannis N. G. (2014). The inflammatory response in myocardial injury, repair, and remodelling. *Nature Reviews Cardiology*.

[59] Prabhu S. D., Frangogiannis N. G. (2016). The biological basis for cardiac repair after myocardial infarction. *Circulation Research*.

[60] Turner N. A. (2016). Inflammatory and fibrotic responses of cardiac fibroblasts to myocardial damage associated molecular patterns (DAMPs). *Journal of Molecular and Cellular Cardiology*.

[61] Tian Y., Piras B. A., Kron I. L., French B. A., Yang Z. (2015). Adenosine 2B receptor activation reduces myocardial reperfusion injury by promoting anti-inflammatory macrophages differentiation via PI3K/Akt pathway. *Oxidative Medicine and Cellular longevity*.

[62] Cronstein B. N., Sitkovsky M. (2017). Adenosine and adenosine receptors in the pathogenesis and treatment of rheumatic diseases. *Nature Reviews Rheumatology*.

[63] Rossello X., Riquelme J. A., Davidson S. M., Yellon D. M. (2018). Role of PI3K in myocardial ischaemic preconditioning: mapping pro-survival cascades at the trigger phase and at reperfusion. *Journal of Cellular and Molecular Medicine*.

[64] Parapanov R., Lugrin J., Rosenblatt-Velin N. (2015). Toll-like receptor 5 deficiency exacerbates cardiac injury and inflammation induced by myocardial ischaemia-reperfusion in the mouse. *Clinical Science*.

[65] Jiménez-Dalmaroni M. J., Gerswhin M. E., Adamopoulos I. E. (2016). The critical role of toll-like receptors - from microbial recognition to autoimmunity: a comprehensive review. *Autoimmunity Reviews*.

[66] Niermann C., Gorressen S., Klier M. (2016). Oligophrenin1 protects mice against myocardial ischemia and reperfusion injury by modulating inflammation and myocardial apoptosis. *Cellular Signalling*.

[67] Pohl J., Hendgen-Cotta U. B., Stock P. (2017). Myocardial expression of macrophage migration inhibitory factor in patients with heart failure. *Journal of Clinical Medicine*.

[68] Pohl J., Hendgen-Cotta U. R., Rammos C. (2016). Targeted intracellular accumulation of macrophage migration inhibitory factor in the reperfused heart mediates cardioprotection. *Thrombosis and Haemostasis*.

[69] Rossello X., Burke N., Stoppe C., Bernhagen J., Davidson S. M., Yellon D. M. (2016). Exogenous administration of recombinant MIF at physiological concentrations failed to attenuate infarct size in a langendorff perfused isolated mouse heart model. *Cardiovascular Drugs and Therapy*.

[70] Chang C., Ji Q., Wu B. (2015). Chemerin15-Ameliorated cardiac ischemia-reperfusion injury is associated with the induction of alternatively activated macrophages. *Mediators of Inflammation*.

[71] Rourke J. L., Dranse H. J., Sinal C. J. (2015). CMKLR1 and GPR1 mediate chemerin signaling through the RhoA/ROCK pathway. *Molecular and Cellular Endocrinology*.

[72] Yue Y., Yang X., Feng K. (2017). M2b macrophages reduce early reperfusion injury after myocardial ischemia in mice: a predominant role of inhibiting apoptosis via A20. *International Journal of Cardiology*.

[73] Afonina I. S., Zhong Z., Karin M., Beyaert R. (2017). Limiting inflammation-the negative regulation of NF-*κ*B and the NLRP3 inflammasome. *Nature Immunology*.

[74] Liu Y., Yang H., Liu L.-X. (2016). NOD2 contributes to myocardial ischemia/reperfusion injury by regulating cardiomyocyte apoptosis and inflammation. *Life Sciences*.

[75] Negroni A., Pierdomenico M., Cucchiara S., Stronati L. (2018). NOD2 and inflammation: current insights. *Journal of Inflammation Research*.

[76] Canna S. W., Goldbach-Mansky R. (2018). Introduction: autoinflammatory syndromes special issue-hidden mysteries in the corners of autoinflammation. *International Immunology*.

[77] Yang C., Jiao Y., Yan N. (2017). NOD2 mediates isoflurane preconditioning-induced protection of myocardial injury. *Neuroscience Letters*.

[78] Szymanski A. M., Ombrello M. J. (2018). Using genes to triangulate the pathophysiology of granulomatous autoinflammatory disease: NOD2, PLCG2 and LACC1. *International Immunology*.

[79] Zhang H., Zhu T., Liu W. (2015). TIPE2 acts as a negative regulator linking NOD2 and inflammatory responses in myocardial ischemia/reperfusion injury. *Journal of Molecular Medicine*.

[80] Goldsmith J. R., Chen Y. H. (2017). Regulation of inflammation and tumorigenesis by the TIPE family of phospholipid transfer proteins. *Cellular and Molecular Immunology*.

[81] Montecucco F., Liberale L., Bonaventura A., Vecchie A., Dallegri F., Carbone F. (2017). The role of inflammation in cardiovascular outcome. *Current Atherosclerosis Reports*.

[82] Arslan F., de Kleijn D., Timmers L., Doevendans P., Pasterkamp G. (2008). Bridging innate immunity and myocardial ischemia/reperfusion injury: the search for therapeutic targets. *Current Pharmaceutical Design*.

[83] Timmers L., Pasterkamp G., de Hoog V. C., Arslan F., Appelman Y., de Kleijn D. P. V. (2012). The innate immune response in reperfused myocardium. *Cardiovascular Research*.

[84] Hausenloy D. J., Garcia-Dorado D., Botker H. E. (2017). Novel targets and future strategies for acute cardioprotection: position paper of the european society of cardiology working group on cellular biology of the heart. *Cardiovascular Research*.

[85] Hamid T., Prabhu S. D. (2017). Immunomodulation is the key to cardiac repair. *Circulation Research*.

[86] Boag S. E., Das R., Shmeleva E. V. (2015). T lymphocytes and fractalkine contribute to myocardial ischemia/reperfusion injury in patients. *Journal of Clinical Investigation*.

[87] Epelman S., Liu P. P., Mann D. L. (2015). Role of innate and adaptive immune mechanisms in cardiac injury and repair. *Nature Reviews Immunology*.

[88] Hofmann U., Frantz S. (2015). Role of lymphocytes in myocardial injury, healing, and remodeling after myocardial infarction. *Circulation Research*.

[89] Bonaventura A., Montecucco F., Dallegri F. (2016). Cellular recruitment in myocardial ischaemia/reperfusion injury. *European Journal of Clinical Investigation*.

[90] Kaplan A., Altara R., Eid A., Booz G. W., Zouein F. A. (2016). Update on the protective role of regulatory T cells in myocardial infarction. *Journal of Cardiovascular Pharmacology*.

[91] Wang Y.-P., Xie Y., Ma H. (2016). Regulatory T lymphocytes in myocardial infarction: a promising new therapeutic target. *International Journal of Cardiology*.

[92] Xia N., Jiao J., Tang T.-T. (2015). Activated regulatory T-cells attenuate myocardial ischaemia/reperfusion injury through a CD39-dependent mechanism. *Clinical Science*.

[93] Ramjee V., Li D., Manderfield L. J. (2017). Epicardial YAP/TAZ orchestrate an immunosuppressive response following myocardial infarction. *Journal of Clinical Investigation*.

[94] Zacchigna S., Martinelli V., Moimas S. (2018). Paracrine effect of regulatory T cells promotes cardiomyocyte proliferation during pregnancy and after myocardial infarction. *Nature Communications*.

[95] Xiao J., Yu K., Li M., Xiong C., Wei Y., Zeng Q. (2017). The IL-2/anti-IL-2 complex attenuates cardiac ischaemia-reperfusion injury through expansion of regulatory T cells. *Cellular Physiology and Biochemistry*.

[96] Fang J., Hu F., Ke D. (2016). *N*-dimethylsphingosine attenuates myocardial ischemia-reperfusion injury by recruiting regulatory T cells through PI3K/Akt pathway in mice. *Basic Research in Cardiology*.

[97] Rizk F. H., Abdel Ghafar M. T., Soliman N. A. (2018). Vildagliptin recruits regulatory T cells in patients undergoing primary percutaneous coronary intervention. *Immunological Investigations*.

[98] Zlatanova I., Pinto C., Silvestre J. S. (2016). Immune modulation of cardiac repair and regeneration: the art of mending broken hearts. *Frontiers in Cardiovascular Medicine*.

[99] Li J., Tan J., Martino M. M., Lui K. O. (2018). Regulatory T-cells: potential regulator of tissue repair and regeneration. *Frontiers in Immunology*.

[100] Goltz D., Huss S., Ramadori E., Büttner R., Diehl L., Meyer R. (2015). Immunomodulation by splenectomy or by FTY720 protects the heart against ischemia reperfusion injury. *Clinical and Experimental Pharmacology and Physiology*.

[101] Rana A., Sharma S. (2016). Mechanism of sphingosine-1-phosphate induced cardioprotection against I/R injury in diabetic rat heart: possible involvement of glycogen synthase kinase 3*β* and mitochondrial permeability transition pore. *Clinical and Experimental Pharmacology and Physiology*.

[102] Luk F. S., Kim R. Y., Li K. (2016). Immunosuppression with FTY720 reverses cardiac dysfunction in hypomorphic ApoE mice deficient in SR-BI expression that survive myocardial infarction caused by coronary atherosclerosis. *Journal of Cardiovascular Pharmacology*.

[103] Hagihara K., Kinoshita K., Ishida K. (2017). A genome-wide screen for FTY720-sensitive mutants reveals genes required for ROS homeostasis. *Microbial Cell*.

[104] Erikson J. M., Valente A. J., Mummidi S. (2017). Targeting TRAF3IP2 by genetic and interventional approaches inhibits ischemia/reperfusion-induced myocardial injury and adverse remodeling. *Journal of Biological Chemistry*.

[105] Das N. A., Carpenter A. J., Yoshida T. (2018). TRAF3IP2 mediates TWEAK/TWEAKR-induced pro-fibrotic responses in cultured cardiac fibroblasts and the heart. *Journal of Molecular and Cellular Cardiology*.

[106] Wang K., Wen S., Jiao J. (2018). IL-21 promotes myocardial ischaemia/reperfusion injury through the modulation of neutrophil infiltration. *British Journal of Pharmacology*.

[107] He S., Wang X., Chen A. (2017). Myocardial ischemia/reperfusion injury: the role of adaptor proteins Crk. *Perfusion*.

[108] Boros D., Thompson J., Larson D. (2016). Adenosine regulation of the immune response initiated by ischemia reperfusion injury. *Perfusion*.

[109] Grilo G. A., Shaver P. R., de Castro Brás L. E. (2017). Mechanisms of cardioprotection via modulation of the immune response. *Current Opinion in Pharmacology*.

[110] Piek A., Du W., de Boer R. A., Silljé H. H. W. (2018). Novel heart failure biomarkers: why do we fail to exploit their potential?. *Critical Reviews in Clinical Laboratory Sciences*.

[111] Lin L., Yang Z., Zheng G. (2018). Analyses of changes in myocardial long non-coding RNA and mRNA profiles after severe hemorrhagic shock and resuscitation via RNA sequencing in a rat model. *BMC Molecular Biology*.

[112] Du M., Huang K., Huang D. (2015). Renalase is a novel target gene of hypoxia-inducible factor-1 in protection against cardiac ischaemia-reperfusion injury. *Cardiovascular Research*.

[113] Jungi S., Fu X., Segiser A. (2018). Enhanced cardiac S100A1 expression improves recovery from global ischemia-reperfusion injury. *Journal of Cardiovascular Translational Research*.

[114] Szczepanek K., Xu A., Hu Y. (2015). Cardioprotective function of mitochondrial-targeted and transcriptionally inactive STAT3 against ischemia and reperfusion injury. *Basic Research in Cardiology*.

[115] Conklin D. J., Guo Y., Jagatheesan G. (2015). Genetic deficiency of glutathione S -transferase P increases myocardial sensitivity to ischemia-reperfusion injury. *Circulation Research*.

[116] Kanwal A., Nizami H. L., Mallapudi S., Putcha U. K., Mohan G. K., Banerjee S. K. (2016). Inhibition of SGLT1 abrogates preconditioning-induced cardioprotection against ischemia-reperfusion injury. *Biochemical and Biophysical Research Communications*.

[117] Khan N. A., Abid M., Ahmad A., Abuzinadah M. F., Basheikh M., Kishore K. (2017). Cardioprotective effect of coenzyme Q10 on apoptotic myocardial cell death by regulation of bcl-2 gene expression. *Journal of Pharmacology and Pharmacotherapeutics*.

[118] Jiang Y., Tian L. L., Wang L. H. (2017). Cardioprotective effects of Serca2a overexpression against ischemiareperfusion- induced injuries in rats. *Current Gene Therapy*.

[119] Surinkaew S., Kumphune S., Chattipakorn S., Chattipakorn N. (2013). Inhibition of p38 MAPK during ischemia, but not reperfusion, effectively attenuates fatal arrhythmia in ischemia/reperfusion heart. *Journal of Cardiovascular Pharmacology*.

[120] McLean B. A., Kienesberger P. C., Wang W. (2013). Enhanced recovery from ischemia-reperfusion injury in PI3K*α* dominant negative hearts: investigating the role of alternate PI3K isoforms, increased glucose oxidation and MAPK signaling. *Journal of Molecular and Cellular Cardiology*.

[121] Chen Z., Zhang X., Liu Y., Liu Z. (2016). Morphine postconditioning protects against reperfusion injury via inhibiting JNK/p38 MAPK and mitochondrial permeability transition pores signaling pathways. *Cellular Physiology and Biochemistry*.

[122] Suchal K., Malik S., Gamad N. (2016). Kaempferol attenuates myocardial ischemic injury via inhibition of MAPK signaling pathway in experimental model of myocardial ischemia-reperfusion injury. *Oxidative Medicine and Cellular Longevity*.

[123] Khan S. I., Malhotra R. K., Rani N. (2017). Febuxostat modulates MAPK/NF-kappaBp65/TNF-alpha signaling in cardiac ischemia-reperfusion injury. *Oxidative Medicine and Cellular Longevity*.

[124] Deng S.-B., Jing X.-D., Wei X.-M. (2017). Triiodothyronine promotes the proliferation of epicardial progenitor cells through the MAPK/ERK pathway. *Biochemical and Biophysical Research Communications*.

[125] Lin B., Xu J., Feng D.-G., Wang F., Wang J.-X., Zhao H. (2018). DUSP14 knockout accelerates cardiac ischemia reperfusion (IR) injury through activating NF-*κ*B and MAPKs signaling pathways modulated by ROS generation. *Biochemical and Biophysical Research Communications*.

[126] Wang Z., Huang H., He W. (2016). Regulator of G-protein signaling 5 protects cardiomyocytes against apoptosis during in vitro cardiac ischemia-reperfusion in mice by inhibiting both JNK1/2 and P38 signaling pathways. *Biochemical and Biophysical Research Communications*.

[127] Zhou Q.-L., Teng F., Zhang Y.-S., Sun Q., Cao Y.-X., Meng G.-W. (2018). FPR1 gene silencing suppresses cardiomyocyte apoptosis and ventricular remodeling in rats with ischemia/reperfusion injury through the inhibition of MAPK signaling pathway. *Experimental Cell Research*.

[128] Shvedova M., Anfinogenova Y., Atochina-Vasserman E. N., Schepetkin I. A., Atochin D. N. (2018). c-Jun N-terminal kinases (JNKs) in myocardial and cerebral ischemia/reperfusion injury. *Frontiers in Pharmacology*.

[129] Filippone S. M., Samidurai A., Roh S. K. (2017). Reperfusion therapy with rapamycin attenuates myocardial infarction through activation of AKT and ERK. *Oxidative Medicine and Cellular Longevity*.

[130] Qin C. X., May L. T., Li R. (2017). Small-molecule-biased formyl peptide receptor agonist compound 17b protects against myocardial ischaemia-reperfusion injury in mice. *Nature Communications*.

[131] Ong S.-B., Hall A., Dongworth R. (2015). Akt protects the heart against ischaemia-reperfusion injury by modulating mitochondrial morphology. *Thrombosis and Haemostasis*.

[132] Mendoza-Torres E., Riquelme J. A., Vielma A. (2018). Protection of the myocardium against ischemia/reperfusion injury by angiotensin-(1–9) through an AT2R and Akt-dependent mechanism. *Pharmacological Research*.

[133] Duan L., Liu C., Hu J. (2018). Epigenetic mechanisms in coronary artery disease: the current state and prospects. *Trends in Cardiovascular Medicine*.

[134] Perrino C., Barabási A.-L., Condorelli G. (2017). Epigenomic and transcriptomic approaches in the post-genomic era: path to novel targets for diagnosis and therapy of the ischaemic heart? position paper of the European society of cardiology working group on cellular biology of the heart. *Cardiovascular Research*.

[135] Virzì G. M., Clementi A., Brocca A., de Cal M., Ronco C. (2018). Epigenetics: a potential key mechanism involved in the pathogenesis of cardiorenal syndromes. *Journal of Nephrology*.

[136] Granger A., Abdullah I., Huebner F. (2008). Histone deacetylase inhibition reduces myocardial ischemia-reperfusion injury in mice. *The FASEB Journal*.

[137] Chistiakov D. A., Orekhov A. N., Bobryshev Y. V. (2017). Treatment of cardiovascular pathology with epigenetically active agents: focus on natural and synthetic inhibitors of DNA methylation and histone deacetylation. *International Journal of Cardiology*.

[138] Schiedel M., Robaa D., Rumpf T., Sippl W., Jung M. (2018). The current state of NAD+ -dependent histone deacetylases (sirtuins) as novel therapeutic targets. *Medicinal Research Reviews*.

[139] Cattelan A., Ceolotto G., Bova S. (2015). NAD+-dependent SIRT1 deactivation has a key role on ischemia-reperfusion-induced apoptosis. *Vascular Pharmacology*.

[140] Hu M.-Z., Zhou B., Mao H.-Y. (2016). Exogenous hydrogen sulﬁde postconditioning protects isolated rat hearts from ischemia/reperfusion injury through sirt1/PGC-1*α* signaling pathway. *International Heart Journal*.

[141] Yang G., Zhang X., Weng X. (2017). SUV39H1 mediated SIRT1 trans-repression contributes to cardiac ischemia-reperfusion injury. *Basic Research in Cardiology*.

[142] Yang G., Weng X., Zhao Y. (2017). The histone H3K9 methyltransferase SUV39H links SIRT1 repression to myocardial infarction. *Nature Communications*.

[143] Han D., Wang J., Ma S., Chen Y., Cao F. (2017). SIRT1 as a promising novel therapeutic target for myocardial ischemia reperfusion injury and cardiometabolic disease. *Current Drug Targets*.

[144] Bochaton T., Crola-Da-Silva C., Pillot B. (2015). Inhibition of myocardial reperfusion injury by ischemic postconditioning requires sirtuin 3-mediated deacetylation of cyclophilin D. *Journal of Molecular and Cellular Cardiology*.

[145] Alam M. R., Baetz D., Ovize M. (2015). Cyclophilin D and myocardial ischemia-reperfusion injury: a fresh perspective. *Journal of Molecular and Cellular Cardiology*.

[146] Nishida Y., Rardin M. J., Carrico C. (2015). SIRT5 regulates both cytosolic and mitochondrial protein malonylation with glycolysis as a major target. *Molecular Cell*.

[147] Wang J., Hu X., Jiang H. (2015). HDAC inhibition: a novel therapeutic target for attenuating myocardial ischemia and reperfusion injury by reversing cardiac remodeling. *International Journal of Cardiology*.

[148] Gillette T. G., Hill J. A. (2015). Readers, writers, and erasers. *Circulation Research*.

[149] Wu Y., Leng Y., Meng Q. (2017). Suppression of excessive histone deacetylases activity in diabetic hearts attenuates myocardial ischemia/reperfusion injury via mitochondria apoptosis pathway. *Journal of Diabetes Research*.

[150] Gidlof O., Johnstone A. L., Bader K. (2016). Ischemic preconditioning confers epigenetic repression of mtor and induction of autophagy through g9a-dependent H3K9 dimethylation. *Journal of the American Heart Association*.

[151] Zhao D., Yang J., Yang L. (2017). Insights for oxidative stress and mTOR signaling in myocardial ischemia/reperfusion injury under diabetes. *Oxidative Medicine and Cellular Longevity*.

[152] Wang P., Fan F., Li X. (2018). Riboflavin attenuates myocardial injury via LSD1-mediated crosstalk between phospholipid metabolism and histone methylation in mice with experimental myocardial infarction. *Journal of Molecular and Cellular Cardiology*.

[153] Li Z., Zhang X., Liu S. (2018). BRG1 regulates NOX gene transcription in endothelial cells and contributes to cardiac ischemia-reperfusion injury. *Biochimica et Biophysica Acta (BBA)-Molecular Basis of Disease*.

[154] Zhang X., Liu S., Weng X. (2018). Brg1 deficiency in vascular endothelial cells blocks neutrophil recruitment and ameliorates cardiac ischemia-reperfusion injury in mice. *International Journal of Cardiology*.

[155] Davidson S. M., Ferdinandy P., Andreadou I. (2019). Multitarget strategies to reduce myocardial ischemia/reperfusion injury. *Journal of the American College of Cardiology*.

